# Supplementary material for: Identification of Host Factors Associated with the Development of Equine Herpesvirus Myeloencephalopathy by Transcriptomic Analysis of Peripheral Blood Mononuclear Cells from Horses
Source: Viruses. 2021 Feb 24;13(3):356. doi: 10.3390/v13030356 (PMC7995974; doi:10.3390/v13030356)
Supplement: Supplementary file 1 [file viruses-13-00356-s001.zip › Supplementary files/Supplementary tables copy.pdf]

Table S1. Mapping summary statistics of mRNA sequencing.			
**Sample ID**	**Total reads**	**Uniquely mapped (number of reads)**	**Uniquely mapped (%)**
EHM_PRE_H1	44184310	37923518	85.8
EHM_POST_H1	40599945	34218921	84.3
EHM_PRE_H2	40137880	34895454	86.9
EHM_POST_H2	47149254	39078943	82.9
EHM_PRE_H3	45889974	39613428	86.3
EHM_POST_H3	39483290	32787239	83.0
EHM_PRE_H4	47767833	41484855	86.9
EHM_POST_H4	38260560	31521306	82.4
EHM_PRE_H5	49226922	42871550	87.0
EHM_POST_H5	40912839	34362844	84.0
EHM_PRE_H7	38847518	33686604	86.7
EHM_POST_H7	38231216	32466324	84.9
EHM_PRE_H9	35120357	30748238	87.6
EHM_POST_H9	42198521	35572673	84.3
EHM_PRE_H15	47675687	40789167	85.6
EHM_POST_H15	39075706	32534599	83.3
CONT_PRE_H16	49819781	42630558	85.6
CONT_POST_H16	39358775	32872086	83.5
CONT_PRE_H26	51958124	44491095	85.6
CONT_POST_H26	53090751	45842652	86.4
CONT_PRE_H27	48454087	41617390	85.9
CONT_POST_H27	38965552	31684566	81.3
CONT_PRE_H28	44791662	38284817	85.5
CONT_POST_H28	43886029	37670974	85.8
CONT_PRE_H34	53040512	45283021	85.4
CONT_POST_H34	38011582	32238800	84.8
CONT_PRE_H35	48119400	41310875	85.9
CONT_POST_H35	40193344	35081080	87.3
**Average**	**43730408**	**37270128**	**85.2**

Table S1. Mapping summary statistics of mRNA sequencing. Total reads after sequencing and the number and percent that uniquely mapped to the EquCab3.0 genome are shown for each sample. "CONT" indicates samples from horses that did not develop EHM, "EHM" indicate samples from horses that did develop EHM, "PRE" indicates samples collected prior to EHV-1 challenge, and "POST" indicates samples collected during peak viremia following EHV-1 challenge.

Table S2. Enriched GO terms for the contrast comparison between EHM and non-EHM horses.								
**ID**	**Description**	**Gene Ratio**	**Bg Ratio**	**pvalue**	**p.adjust**	**qvalue**	**Gene ID**	**Gene Count**
**Upregulated**								
GO:0006885	regulation of pH	3/21	73/13991	0.000169	0.009644	0.007029	NOX1/SLC9B1/SLC4A9	3
GO:0002830	positive regulation of type 2 immune response	2/21	14/13991	0.000193	0.009644	0.007029	RSAD2/IL6	2
GO:0001819	positive regulation of cytokine production	5/21	368/13991	0.000176	0.009644	0.007029	NOX1/RSAD2/IL1RL1/IL6/LPL	5
GO:0048661	positive regulation of smooth muscle cell proliferation	3/21	83/13991	0.000248	0.010829	0.007893	NOX1/TGM2/IL6	3
GO:0010883	regulation of lipid storage	2/21	41/13991	0.001699	0.035978	0.026223	IL6/LPL	2
**Downregulated**								
GO:0050900	leukocyte migration	13/106	349/13991	2.28E-06	0.005963	0.005215	DAPK2/CCL5/CX3CR1/CD244/KLRK1/ MMP9/TBX21/PLCB1/DUSP1/SLC7A11/ NLRP12/PDGFB/TREM1	13
GO:0070372	regulation of ERK1 and ERK2 cascade	11/106	278/13991	8.18E-06	0.010703	0.00936	CCL5/FGFR1/INSR/JUN/DUSP1/ATF3/ EPHA4/DUSP6/NLRP12/PDGFB/KLF4	11
GO:0007611	learning or memory	9/106	231/13991	6.39E-05	0.022372	0.019565	CX3CR1/FOS/PLK2/PLCB1/MAP1A/ INSR/JUN/EGR2/SLC7A11	9
GO:0071276	cellular response to cadmium ion	4/106	25/13991	3.48E-05	0.022372	0.019565	MMP9/FOS/JUN/GSN	4
GO:0033002	muscle cell proliferation	8/106	187/13991	8.66E-05	0.022664	0.01982	CCL5/MMP9/FGFR1/ABCC4/IFNG/ JUN/PDGFB/KLF4	8
GO:2001057	reactive nitrogen species metabolic process	5/106	68/13991	0.000162	0.023245	0.020328	CX3CR1/KLRK1/INSR/IFNG/KLF4	5
GO:0046209	nitric oxide metabolic process	5/106	67/13991	0.000151	0.023245	0.020328	CX3CR1/KLRK1/INSR/IFNG/KLF4	5
GO:0051099	positive regulation of binding	7/106	166/13991	0.000265	0.0278	0.024312	BAMBI/MMP9/PLK2/IFNG/EPHA4/ KLF4/DACT1	7
GO:0046777	protein autophosphorylation	8/106	220/13991	0.000265	0.0278	0.024312	DAPK2/PTK6/FGFR1/INSR/JUN/ EPHA4/NLRP12/PDGFB	8

GO:0061614	pri-miRNA transcription by RNA polymerase II	4/106	42/13991	0.000279	0.028111	0.024584	FOS/JUN/PDGFB/KLF4	4
GO:0006066	alcohol metabolic process	9/106	302/13991	0.000475	0.035311	0.03088	SCD/LIPE/CD244/FGFR1/ACER2/NUDT4/PLCB1/IFNG/APOBR	9
GO:0042136	neurotransmitter biosynthetic process	5/106	90/13991	0.000599	0.037325	0.032641	CX3CR1/KLRK1/INSR/IFNG/KLF4	5

Table S2. Enriched GO terms for the contrast comparison between EHM and non-EHM horses. GO term enrichment was performed on the up and down regulated gene lists generated from the between group (contrast) comparison. Upregulated terms are those based on the genes upregulated in EHM horses compared to non-EHM horses. Downregulated terms are those based on the genes downregulated in EHM horses compared to non-EHM horses.

Table S3: Differentially expressed genes unique to non-EHM horses				
**Symbol**	**Log fold change**	**FDR**	**PANTHER family/subfamily**	**Function - UniProtKB (*homo sapiens* ortholog)**
**Upregulated genes**				
BFSP2	2.2	1.5E-03	Phakinin (PTHR23239:SF32)	Required for the correct formation of lens intermediate filaments as part of a complex composed of BFSP1, BFSP2 and CRYAA.
NFE2	2.0	1.5E-02	Transcription factor NF-E2 45 KDA subunit (PTHR24411:SF26)	Component of the NF-E2 complex essential for regulating erythroid and megakaryocytic maturation and differentiation.
ENSECAG00000021212	1.6	3.8E-02	C-type lectin domain family 4 member M (PTHR22802:SF197)	Human ortholog unavailable.
HEY1	1.5	3.0E-03	Hairy/enhancer-of-split related with YRPW motif protein 1 (PTHR10985:SF78)	Transcriptional repressor which binds preferentially to the canonical E box sequence 5'-CACGTG-3'. Downstream effector of Notch signaling required for cardiovascular development. Specifically required for the Notch-induced endocardial epithelial to mesenchymal transition.
CILP	1.4	4.6E-02	Cartilage intermediate layer protein 1 (PTHR15031:SF3)	Probably plays a role in cartilage scaffolding. May act by antagonizing TGF-beta1 (TGFB1) and IGF1 functions.
SCD	1.4	6.1E-12	Acyl-CoA desaturase (PTHR11351:SF73)	Stearyl-CoA desaturase that utilizes O_2 and electrons from reduced cytochrome b5 to introduce the first double bond into saturated fatty acyl-CoA substrates. Plays an important role in body energy homeostasis.
CISH	1.4	2.2E-03	Cytokine-inducible SH2-containing protein (PTHR10155:SF9)	SOCS family proteins form part of a classical negative feedback system that regulates cytokine signal transduction. CIS is involved in the negative regulation of cytokines that signal through the JAK-STAT5 pathway such as erythropoietin, prolactin and interleukin 3 (IL3) receptor. Inhibits STAT5 trans-activation by suppressing its tyrosine phosphorylation.

TCF7L1	1.3	1.8E-02	Transcription factor 7-like 1 (PTHR10373:SF25)	Participates in the Wnt signaling pathway. Binds to DNA and acts as a repressor in the absence of CTNNB1, and as an activator in its presence.
ENSECAG00000009762	1.3	8.5E-04	Carcinoembryonic antigen-related cell adhesion molecule 1 (PTHR44427:SF1)	No human ortholog available.
DUSP6	1.3	1.4E-02	Dual specificity protein phosphatase 6 (PTHR10159:SF45)	Inactivates MAP kinases. Has a specificity for the ERK family. Promotes cell differentiation by regulating MAPK1/MAPK3 activity and regulating the expression of AP1 transcription factors.
FAM111B	1.2	8.6E-03	Protein FAM111B (PTHR14389:SF4)	No function listed.
ENSECAG00000022644	1.1	1.7E-02	C-type lectin-like domain family 1 (PTHR46746:SF4)	No human ortholog available.
FRMD4A	1.1	1.1E-02	FERM domain-containing protein 4A (PTHR46079:SF3)	Scaffolding protein that regulates epithelial cell polarity by connecting ARF6 activation with the PAR3 complex. Plays a redundant role with FRMD4B in epithelial polarization.
ENSECAG00000019430	1.1	1.8E-04	Apolipoprotein L2 (PTHR14096:SF27)	No human ortholog available.
LZTS1	1.1	4.1E-02	Zipper putative tumor suppressor 1-related (PTHR19354:SF5)	Involved in the regulation of cell growth. May stabilize the active CDC2-cyclin B1 complex and thereby contribute to the regulation of the cell cycle and the prevention of uncontrolled cell proliferation.
SHISA5	1.0	1.3E-02	Protein SHISA-5 (PTHR31395:SF14)	Can induce apoptosis in a caspase-dependent manner and plays a role in p53/TP53-dependent apoptosis.
**Downregulated genes**				
CGA	-1.2	2.8E-05	Glycoprotein hormones alpha chain (PTHR11509:SF0)	Shared alpha chain of the active heterodimeric glycoprotein hormones thyrotropin/thyroid stimulating hormone/TSH, lutropin/luteinizing hormone/LH, follitropin/follicle stimulating hormone/FSH and choriogonadotropin/CG. These hormones bind specific receptors on target cells that in turn activate downstream signaling pathways.

SYTL2	-1.0	2.2E-03	Synaptotagmin-like protein 2 (PTHR45716:SF5)	Isoform 1 acts as a RAB27A effector protein and plays a role in cytotoxic granule exocytosis in lymphocytes. It is required for cytotoxic granule docking at the immunologic synapse.
WWTR1	-1.0	7.0E-03	WW domain-containing transcription regulator protein 1 (PTHR17616:SF6)	Transcriptional coactivator which acts as a downstream regulatory target in the Hippo signaling pathway that plays a pivotal role in organ size control and tumor suppression by restricting proliferation and promoting apoptosis. Regulates embryonic stem-cell self-renewal, promotes cell proliferation and epithelial-mesenchymal transition.

Table S3. Differentially expressed genes unique to non-EHM horses. Differentially expressed genes (adjusted p value < 0.05 and log2 fold change $> |1|$) are displayed here. Upregulated refers to genes upregulated during viremia compared to pre-challenge and downregulated refers to genes downregulated during viremia compared to pre-challenge. The PANTHER family and subfamily category is listed for the equine gene [57] and the UniProtKB function for the protein associated with the orthologous human gene is shown [58].

Table S4. Mapping summary statistics for miRNA			
**Sample ID**	**Total reads**	**Mapped reads (number of reads)**	**Mapped reads (%)**
EHM_PRE_H1	14579372	8401412	57.6
EHM_POST_H1	16138773	10132028	62.8
EHM_PRE_H2	27603885	18045362	65.4
EHM_POST_H2	10003035	6177560	61.8
EHM_PRE_H3	16196518	9932650	61.3
EHM_POST_H3	13411346	7434254	55.4
EHM_PRE_H4	14230204	8689707	61.1
EHM_POST_H4	13378608	8204075	61.3
EHM_PRE_H5	11389204	7010810	61.6
EHM_POST_H5	12190031	6308410	51.8
EHM_PRE_H7	6538519	3809812	58.3
EHM_PRE_H9	8758431	5622680	64.2
EHM_POST_H7	11022200	6965796	63.2
EHM_POST_H9	17781885	11311463	63.6
EHM_PRE_H15	13441490	8441676	62.8
EHM_POST_H15	10565453	6343105	60
CONT_PRE_H16	18666363	10063969	53.9
CONT_POST_H16	16333781	9902090	60.6
CONT_PRE_H26	12620476	7639817	60.5
CONT_POST_H26	20738238	13052381	62.9
CONT_PRE_H27	14274791	8648212	60.6
CONT_POST_H27	14478575	9121947	63
CONT_PRE_H28	13958536	8185342	58.6
CONT_POST_H28	11650722	6770100	58.1
CONT_PRE_H34	16414130	8970337	54.7
CONT_POST_H34	14229914	7987735	56.1
CONT_PRE_H35	12118767	6341040	52.3
CONT_POST_H35	12721783	7562530	59.4
**Average**	**14122680**	**8467011**	**59.7**

Table S4. Mapping summary statistics of miRNA sequencing. Total reads after sequencing and the number and percent that uniquely mapped to the combined equine and viral genomes are shown for each sample. “CONT” indicates samples from horses that did not develop EHM, “EHM” indicate samples from horses that did develop EHM, “PRE” indicates samples collected prior to EHV-1 challenge, and “POST” indicates samples collected during peak viremia following EHV-1 CH.
